# Insights into financial technology (FinTech): a bibliometric and visual study

**DOI:** 10.1186/s40854-021-00285-7

**Published:** 2021-10-06

**Authors:** Bo Li, Zeshui Xu

**Affiliations:** 1 College of Statistics and Mathematics, Zhejiang Gongshang University, Hangzhou, 610064 China; 2 Business School, Sichuan University, Chengdu, 610064 China

**Keywords:** Bibliometric analysis, Citation structure, Development trends, FinTech, Visualization networks

## Abstract

This paper conducted a comprehensive analysis based on bibliometrics and science mapping analysis. First, 848 publications were obtained from Web of Science. Their fundamental characteristics were analyzed, including the types, annual publications, hot research directions, and foci (by theme analysis, co-occurrence analysis, and timeline analysis of author keywords). Next, the prolific objects (at the level of countries/regions, institutions, journals, and authors) and corresponding pivotal cooperative relationship networks were used to highlight who pays attention to FinTech. Furthermore, the citation structures of authors and journals were investigated, including citation and co-citation. Additionally, this paper presents the burst detection analysis of cited authors, journals, and references. Finally, combining the analysis results with the current financial environment, the challenges and future development opportunities are discussed further. Accordingly, a comprehensive study of the FinTech documents not only reviews the current research characteristics and trajectories but also helps scholars find the appropriate research entry point and conduct in-depth research.

## Introduction

FinTech (abbreviation for financial technology, as an emerging technical term) is driven by a variety of emerging frontier technologies. It is a series of new business models, new technology applications, and new products and services that have a significant impact on the financial market and supply of financial services. It has attracted wide attention because of the following advantages: improving the efficiency of operations, reducing operating costs effectively, disrupting the existing industry structures, blurring industry boundaries, facilitating strategic disintermediation, providing new gateways for entrepreneurship, and democratizing access to financial services (Agarwal and Zhang 2020; Cao et al. 2020; Admati and Hellwig 2013; Loubere 2017; Pinochet et al. 2019; Philippon 2016; Yang et al. 2020; Suryono et al. 2020). The key technologies of FinTech include internet technology (including Internet and Web of Things) (Ruan et al. 2019), big data (Chen et al. 2017; Gai et al. 2018a), artificial intelligence (Belanche et al. 2019), distributed technology (blockchain and cloud computing) (Belanche et al. 2019; Gomber et al. 2018; Chen et al. 2019; Wamba et al. 2020; Miau and Yang 2018), and security technology (biometric technology) (Gai et al. 2018a, b; Wamba et al. 2020). Under the influence of these technologies, the traditional development model of the financial industry has changed.

Furthermore, scholars have done studies involving theories and applications. To examine FinTech adoption and use from the technology acceptance perspective, Singh et al. (2020) proposed a research framework by adding substructures of the technology acceptance model. The FinTech ecosystem consisting of FinTech startups, technology developers, government, financial customers, and traditional financial institutions was presented by Lee and Shin (2018). Accordingly, the application of FinTech has been involved in many areas, such as mobile payment (Gomber et al. 2018), mobile networks (Gai et al. 2016; Wen et al. 2013; Zhang et al. 2013; Zhang and Soong 2004), big data (Yin and Gai 2015), blockchain (Wamba et al. 2020; Iman 2018), P2P lending (Gomber et al. 2018; Ge et al. 2017; Suryono et al. 2021; Wang et al. 2020a, b), cloud computing (Castiglione et al. 2015; Gai et al. 2018a, b), banking service, investment funds, retail groups, and telecom operators (Singh et al. 2020), image processing (Castiglione et al. 2007), and data analysis techniques (Qiu et al. 2015).

FinTech promotes the development of the financial industry. Specifically, it will be easier to collect and analyze data in the financial market to reduce information asymmetry. Trading and investment strategies based on artificial intelligence and big data can redefine the price discovery mechanism of the financial market and improve transaction speed, promoting the liquidity of the financial market and enhancing the efficiency and stability of the financial market. Regulators analyze, warn, and prevent systemic risks in the financial market more efficiently. Additionally, the smart FinTech helps save labor costs and reduce staff duplication by combining big data with artificial intelligence. Next, the development and application of FinTech help more people, especially the poor, obtain financial services at a lower cost and more conveniently, and share more reform results. Moreover, because of the “Belt and Road”, many countries share the achievements of FinTech. For example, our country’s mobile payment helps the economic and financial development of countries along the “Belt and Road”.

To explore the boundaries and research paradigms of the financial disciplines that have been broken and reconstructed, this paper analyzed the current research characteristics and development trends according to the publications in the field of FinTech. 95.28% of all publications were published after 2015 (according to Web of Science (WoS)). The explosive growth and the advantages of bringing great convenience to economic management activities have prompted us to conduct a comprehensive analysis and explore the current challenges and opportunities facing the field of FinTech. It is essential for scholars who are interested in this field to conduct better and more in-depth research. Additionally, a comprehensive analysis helps investigate the development track characteristics and disclose statistical patterns through bibliometric analysis (Borgman and Furner 2002; Wang et al. 2018). Furthermore, this paper investigated the current research hot topics, identified the challenges, and predicted the future development trends.

Bibliometrics, as a statistical and quantitative analysis of academic literature, has access to visualizing the analysis results using science mapping analysis tools, such as CiteSpace and Vosviewer (Chen 2006; Stopar and Bartol 2019; Van Eck and Waltman 2010), thereby improving the readability of analysis results. Bibliometric analysis has been widely applied in different research areas, such as bitcoin (Merediz-Solà and Bariviera 2019), blockchain (Miau and Yang 2018), fuzzy decision making (Liu and Liao 2017), deep learning (Li et al. 2020), social sciences (Nasir et al. 2020), business and economics (Merigo et al. 2016), COVID-19 (Lou et al. 2020), financial innovation (Li and Xu 2021), poverty cycles (Qin et al. 2021), blockchain and cryptocurrency (Nasir et al. 2021), and journals (*European Journal of Operational Research* (Laengle et al. 2017), *Information Sciences* (Yu et al. 2017), *IEEE Transaction on Fuzzy Systems* (Yu et al. 2018), *Environmental Impact Assessment Review* (Nita 2019), and *International Journal of Systems Science* (Wang et al. 2021)). The two visualization tools, i.e., CiteSpace and Vosviewer, can assist the bibliometric method in revealing the static and dynamic characteristics of FinTech publications from various aspects. For example, the co-occurrence network of author keywords demonstrates the main research topics; the citation and co-citation analysis highlight the top influential objects; the burst detection analysis and timeline view can exhibit changes in a certain period. These processes are called science mapping analysis (Van Eck and Waltman 2010; Cobo et al. 2011).

The contributions of this paper can be summarized as follows: (1) Illustrate the basic features of FinTech publications, including the types, annual publications, main research directions by co-occurrence analysis of keywords, and dynamic changes of research focus by timeline analysis; (2) Explore popular countries/regions, institutions, journals, and authors and the collaboration relationship networks, and present the citation and co-citation networks to highlight the influential authors and journals; (3) Furthermore, detect the dynamic changes of cited authors, cited journals and cited references based on burst detection analysis, and more intuitively show the citation process of all FinTech publications based on overlay analysis; (4) With the current special environment, discuss the challenges FinTech faced and future possible development directions.

The rest of this paper is organized as follows: “Data and methods” section briefly describes the data and methods used in this paper. “Fundamental characteristics of FinTech publications” section presents the foundation characteristics of all FinTech publications, in terms of types, annual publications, current research directions and themes, co-occurrence, and timeline analysis of author keywords. The top productive countries/regions, institutions, and journals are presented in “Productive object analysis and cooperation relationship analysis” section. Additionally, the cooperation relationship is demonstrated. “Citation structure analysis” section investigates the citation structure, including citation and co-citation of authors and journals, respectively. Meanwhile, a burst detection analysis of cited journals, cited authors, and cited references is conducted. Furthermore, the current challenges and future possible research directions are discussed in “Discussion” section. “Conclusions” section ends this paper with some conclusions.

## Data and methods

The literature data used in this paper are obtained from WoS (Falagas et al. 2008), one of the most widely used databases in academics, owned by Thomson Reuters Corporation. In this paper, we derived data through the search function in WoS by selecting as Database = Web of Science ™ Core Collection database; Topic search = FinTech or “Financial technology” or “Financial technologies”; Timespan = 1900–2020 (The data were derived on September 23, 2020. We searched the documents from the earliest time of WoS). As a result, 848 documents were retrieved and exported in plain text file format for software (CiteSpace and Vosviewer) bibliometric analysis. The contents in the derived documents are representative, including title, abstract, keywords, citations and references.

**Fig. 1:**
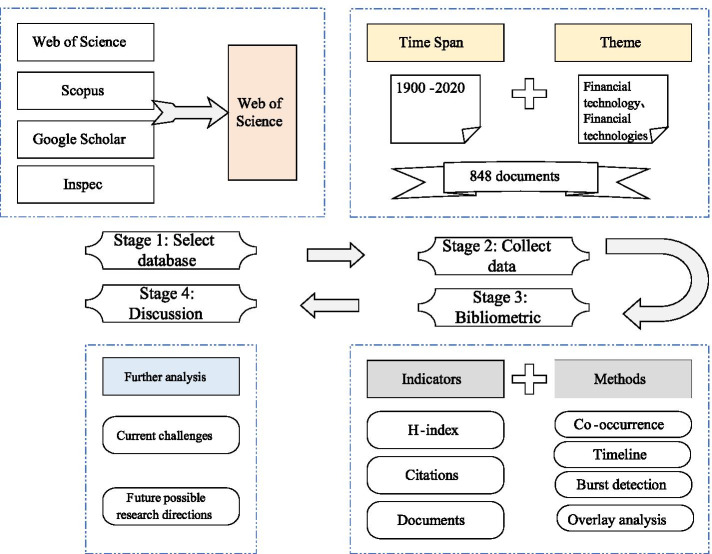
The research framework and process of this study

As presented in the Introduction, bibliometrics is used to highlight the development trajectory and characteristics of a particular research field (Mourao and Martinho 2020). This paper used the bibliometric analysis method to evaluate the development of FinTech documents from the following aspects: (1) start with the types and annual publications with significant indexes (such as numbers and rates), the distribution among different countries/regions, and important branches. Moreover, the productive countries/regions, institutions, journals, and authors are assessed by several recognized indicators, including the total number of publications (TP), the total number of citations (TC), the average citations per publications (AC), and H-index. (2) With statistics and visualization tools, the science mapping analysis is conducted to deeply master the characteristics of FinTech documents, such as the dynamic development trend. CiteSpace and Vosviewer, as two mature visualization tools, effectively illustrate the inner relationship of documents and visualize them in different ways, such as clustering and dynamic timeline (Chen 2006; Stopar and Bartol 2019; Kou et al. 2014). Through several bibliometric methods, including co-occurrence analysis, timeline analysis, burst detection and co-authorship analysis, this paper presented the keyword situation, citations, and cooperation networks of countries/regions and institutions on FinTech research. The whole process of bibliometric analysis in the field of FinTech can be illustrated in Fig. 1.

## Fundamental characteristics of FinTech publications

### Types and annual publications

**Fig. 2:**
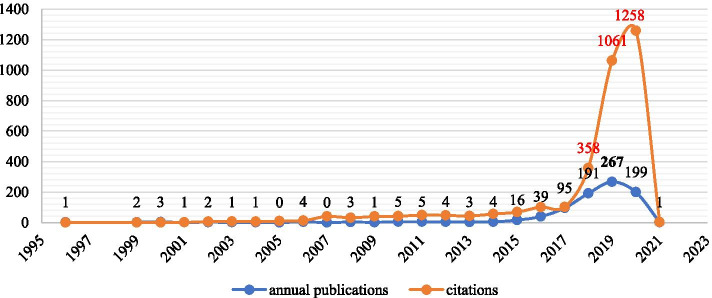
The annual publications and citations

For the 848 obtained publications in the field of FinTech, the first document was written by Ronner and Trappeniers (1996), that is, *Currency exposure management within Philips*, a proceedings paper. The average number of publications is 33, which is low because a lot of literature has exploded in the past five years. The types of the 848 documents and annual publications are presented in Table 1 and Fig. 2, respectively.

**Table 1:** Types of all 848 documents

Types	Numbers	Rate/848 (%)
Article	531	63.139
Proceedings Paper	236	28.062
Early Access	51	6.064
Review	35	4.162
Editorial Material	33	3.924
Book Review	10	1.189
Book Chapter	4	0.476
Correction	2	0.238
Data Paper	1	0.119
Meeting Abstract	1	0.119

From Table 2, most publications are articles with 531, accounting for 63.139%. Followed, 236 are proceedings papers, accounting for 28.062%; 51 (6.064%) are early access. Besides, there are 35 reviews, 33 editorial materials, 10 book reviews, four book chapters, two corrections, one data paper, and one meeting abstract. Articles and proceedings papers dominated the FinTech publications and accounted for 91.201%. Figure 1 illustrates the annual publications and the number of citations per year. 2019 has the greatest number of publications with 267 and ranks second for the numbers of citations with 1,061. 2020 and 2018 have 199 and 191 publications, respectively. 2020 has the greatest number of citations with 1,258. Furthermore, the H-index (Wang et al. 2020a, b) of all documents is 27 and total citations are 3,338 (remove self-citations: 2,423). All the above phenomena reflect that FinTech is a relatively new field and has attracted wide attention recently. In contrast, it reflects that there is still room for development.

**Table 2:** The top 5 most productive countries/regions

Rank	Country/region	TP	TC	AC	≥ 100	≥ 50	H-index	The most cited article	Time cited
1	USA	159	1,235	7.77	2	4	18	Berger, AN (2003)	158
2	China	146	566	3.88	0	0	13	Gai, K et al. (2018)	46
3	England	96	491	5.11	1	2	11	Gabor, D and Brooks, S (2017)	64
4	Australia	50	237	4.74	0	0	9	Arner DW et al. (2017)	38
5	Russia	50	24	0.48	0	0	2	Kostin GA et al. (2017)	9

**Fig. 3:**
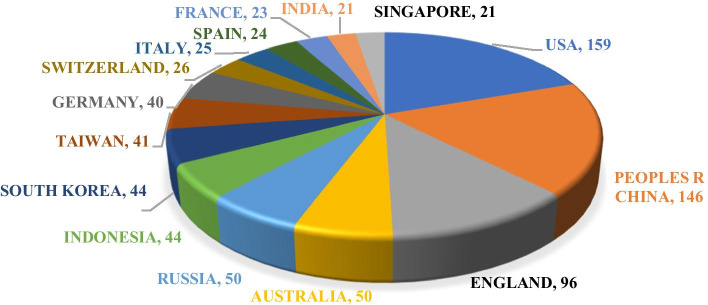
The countries/regions with more than 20 publications

Figure 3 demonstrates the countries/regions with more than 20 publications. Taiwan, a part of China, is studied as a region in this paper. As shown in Fig. 3, the United States is the most productive country with 159. Together with Taiwan, China ranks first with 187. Then, the third to the tenth are England (96), Australia (50), Russia (50), Indonesia (44), South Korea (44), Taiwan (41), Germany (40), and Switzerland (26), respectively. The top 10 countries/regions account for 82.076% of the 848 publications. The United States and China, as the top two most productive countries, have 305 publications, accounting for 35.967%.

### Research directions and themes

**Fig. 4:**
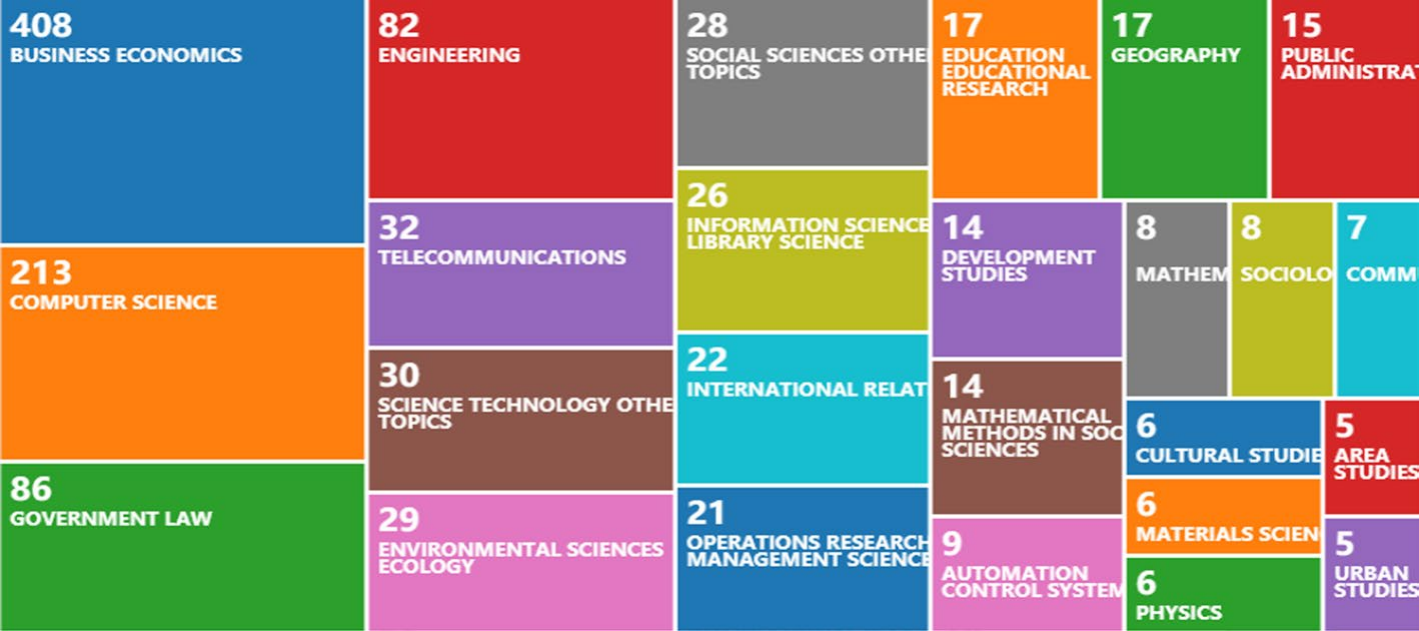
The top 25 hot research directions (generated using WoS on data)

**Fig. 5:**
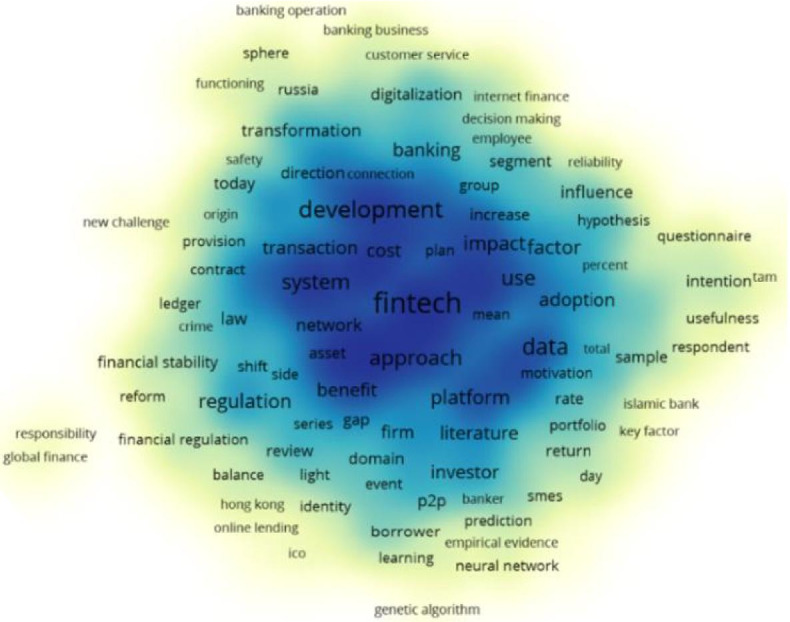
The theme of all 848 publications (generated using Vosviewer on data)

**Fig. 6:**
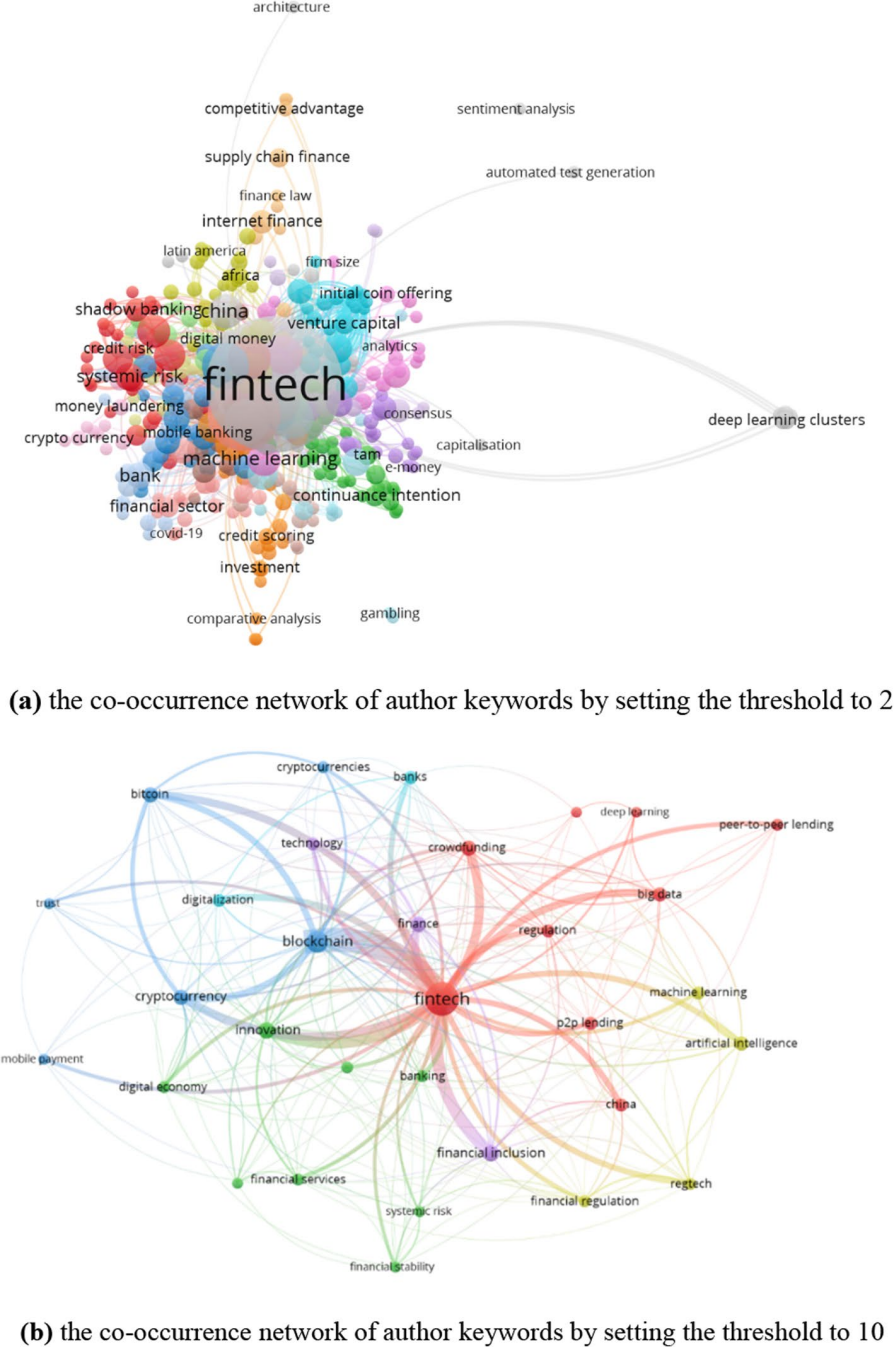
The co-occurrence network of author keywords (generated using Vosviewer on data)

The top 25 research directions of the publications are shown in Fig. 4. Business economics and computer science are the most popular research directions. The number of publications for business economics is 408, and this accounts for 48.11%. The number of publications for computer science is 213, which accounts for 25.12%. Furthermore, research is widespread in government law (86), engineering (82), telecommunications (32), science technology and other topics (30), and environmental sciences ecology (29). FinTech covers many areas and promotes the development of numerous research directions. Based on Vosviewer, the visualization of the theme of the 848 publications is shown in Fig. 5. We can see that, except for FinTech, some terms (e.g., data, market, model, system, bank, and neural network) have a high frequency. Moreover, Fig. 6a presents the co-occurrence view of author keywords in this field. The co-occurrence method was first provided in the 1980s, which has been widely applied in bibliometrics or other fields and helps scholars grasp the study hotspots (Ding et al. 2001). We obtained 389 author keywords by setting the minimum number of occurrences of a keyword to 2 and merging financial technology and financial technologies into FinTech. The high-frequency keywords with the close co-occurrence relationship in the field of FinTech are shown in Fig. 6b by setting the minimum number of occurrences of a keyword to 10.

In Fig. 6a, the nodes denote the author keywords, and the sizes mean the occurrences. The link between two keywords reflects that the two keywords appear in one paper simultaneously. The thickness of the lines between nodes means the number of co-occurrences of the two keywords. Specifically, the frequency of “FinTech” is 385 with 338 links (338 keywords appear with it) and 1,106 total link strength (a total of 1,106 times). “blockchain” has 98 occurrences and ranks second, followed by “financial inclusion” with 36, “innovation” with 36, “cryptocurrency” with 34, and “bitcoin” with 29. For “FinTech”, “blockchain” has the strongest connection with it (link strength is 57). Also, keywords that are closely related to “FinTech” include financial inclusion (link strength is 29), innovation (27), crowdfunding (22), and big data (18). In Fig. 6b, we obtained 32 keywords. Popular topics include digitalization (the frequency is 21), machine learning (17), deep learning (11), and Internet finance (8).

### Timeline view of author keywords

**Fig. 7:**
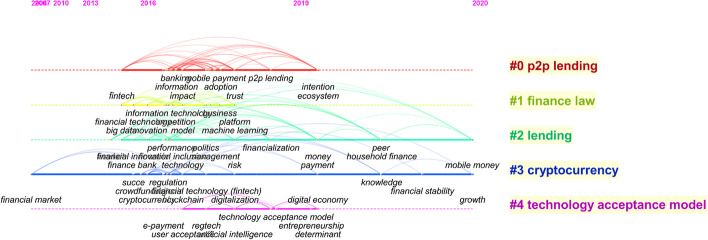
The timeline view of keywords (generated using CiteSpace on data)

As shown in Fig. 6, various keywords belong to different subareas. To understand the dynamic development trend further, this subsection illustrates their timeline view (Fig. 7). From this view, all keywords are classified into 5 clusters, i.e., P2P lending, finance law, lending, cryptocurrency, and technology acceptance model. Cluster 3 is the category with the longest time. The vast majority of keywords broke out after 2014. From 2014 to 2019, research topics, such as banking, mobile payment, P2P lending, financial regulation, e-payment, and big data emerged and continued. Digital economy, household finance, and financial stability were proposed in 2019. Mobile money appeared in 2020. The phenomena verify that FinTech is a new and hot field in recent years once again. With the rapid development of science and technology, much research will explode in a short time. Thus, it is necessary to summarize the research results on time, which highlights the significance of this paper.

## Productive object analysis and cooperation relationship analysis

This section presents the most productive countries/regions, institutions, journals, and authors. Knowing their foci and situations help scholars locate authoritative academics and journals accurately. Moreover, as one important indicator, cooperation relationship analysis is another research point in this section.

### The most productive countries/regions

Table 2 lists the top 5 most productive countries/regions. Some bibliometric indicators, such as TP, TC, AC, the number of publications that are cited equal to or more than 100/50 (≥ 100/ ≥ 50), and H-index are used to demonstrate the citation impact of the productive countries/regions.

The United States has the greatest number of citations with 1,235 and H-index with 18. The most cited US paper is the work of AN Berger *The economic effects of technological progress: Evidence from the banking industry*, a review, published in the *Journal of Money Credit and Banking* (its impact factor is 1.355, belongs to Q3). The paper examined technological progress and its effects on the banking industry (Berger 2003). China has 566 citations. The most cited Chinese paper is the work of KK Gai et al. *A survey on FinTech*, published in the *Journal of Network and Computer Applications* (its impact factor is 5.57, belongs to Q1). It produced a survey of FinTech by collecting and reviewing contemporary achievements and then proposed a theoretical data-driven FinTech framework (Gai et al. 2018a, b). Table 3 lists the top 10 most frequent author keywords of the top 3 most productive countries (i.e., the United States, China, and England). Blockchain is one of the most common research topics, and the frequencies for each country are 13, 17, and 7, respectively. Furthermore, scholars in the United States and China pay more attention to P2P lending, financial inclusion, and cryptocurrency. Regulatory technology (RegTech) is another key topic for Chinese and British scholars. In summary, current hot research directions include blockchain, P2P lending, big data, financial inclusion, and regulation.

**Table 3:** The author keywords of the top 3 productive countries/regions

USA		China		England	
Keywords	Freq	Keywords	Freq	Keywords	Freq
fintech	67	fintech	75	fintech	44
blockchain	13	blockchain	17	financial inclusion	11
p2p lending	8	china	12	blockchain	7
big data	8	p2p lending	12	financial regulation	7
crowdfunding	8	financial inclusion	8	regtech	7
finance	7	internet finance	7	china	5
financial inclusion	6	cryptocurrency	6	finance	5
cryptocurrency	5	mobile payment	6	bitcoin	4
machine learning	5	regtech	6	deep learning clusters	4
innovation	4	crowdfunding	4	genetic learning	4

**Fig. 8:**
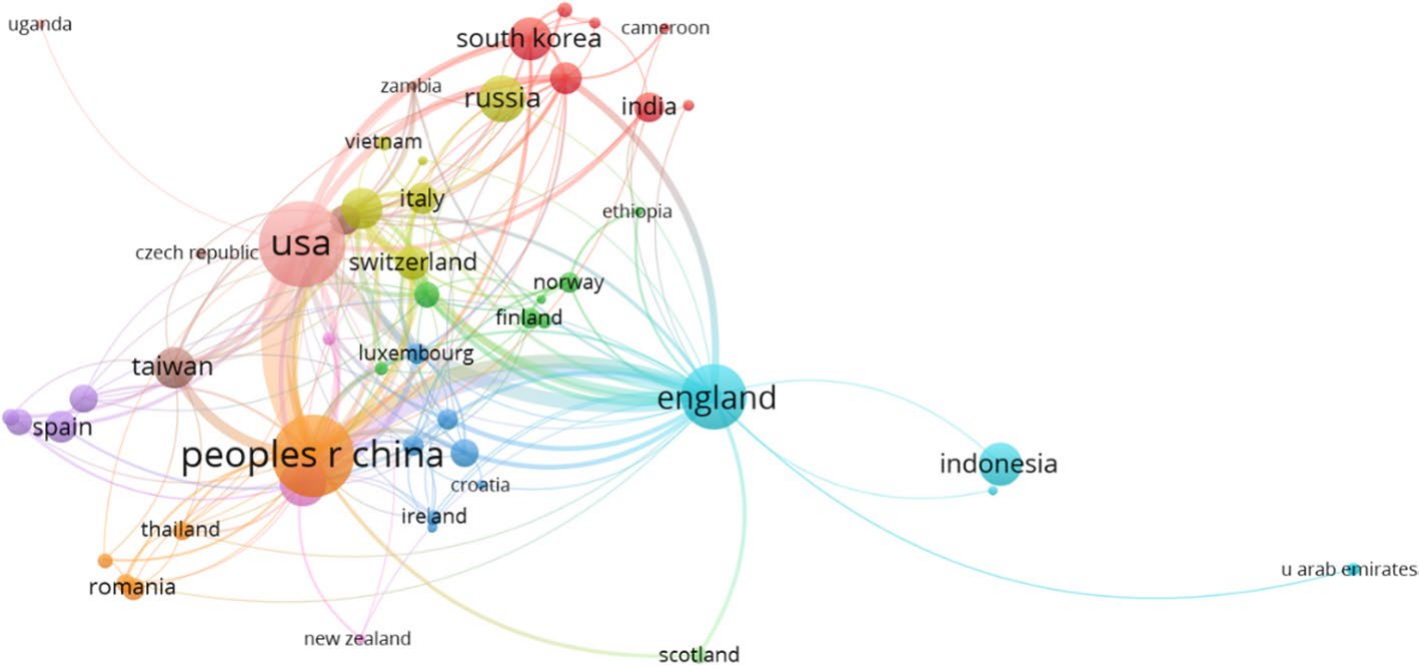
The collaboration network of countries/regions (generated using Vosviewer on data)

The cooperation relationship among countries/regions is illustrated in Fig. 8. There are a total of 86 countries/regions, and we selected 62 terms by setting the minimum number of documents of a country to 2 and presented the closest cooperation network, including 52 terms. They are divided into 11 clusters, and different colors represent different categories. In Fig. 8, the nodes represent the countries/regions, and the sizes of the nodes denote the number of documents. The links between two nodes denote that they have a cooperative relationship with each other. The thicker the link is, the greater their collaboration. The most cooperative countries/regions in each category are the United States, China, England, Russia, Australia, Canada, and South Korea, respectively. The United States is the most cooperative and often cooperates with China, which can be reflected by the links between them. From the perspective of TP and TC of the United States and China, the importance of cooperation is clear.

### Productive institutions

Table 4 exhibits the top 5 most productive institutions. In the list, the University of London has the greatest number of TP, and it is the only institution that has published more than 50 citations. The Ministry of Education Science of Ukraine, University of New South Wales Sydney, University of Hong Kong, and Massachusetts Institute of Technology follow. In terms of TC, the University of New South Wales Sydney ranks first with 148. The second to the fifth institutions are the University of London (111), University of Hong Kong (77), Massachusetts Institute of Technology (72), and Ministry of Education Science of Ukraine (6). The University of New South Wales Sydney and the University of Hong Kong jointly published an article, *FinTech, RegTech, and the Reconceptualization of Financial Regulation* (Arner et al. 2017). It is the most cited publication in the field of FinTech. The phenomenon drives us to explore the cooperative relationship among institutions.

**Table 4:** The top 5 most productive countries/regions

Rank	Country/region	TP	TC	AC	≥ 100	≥ 50	H-index
1	University of London	14	111	7.93	0	1	5
2	Ministry of Education Science of Ukraine	13	6	0.46	0	0	1
3	University of New South Wales Sydney	12	148	11.38	0	0	7
4	University of Hong Kong	12	77	6.42	0	0	4
5	Massachusetts Institute of Technology	10	72	7.2	0	0	3

**Fig. 9:**
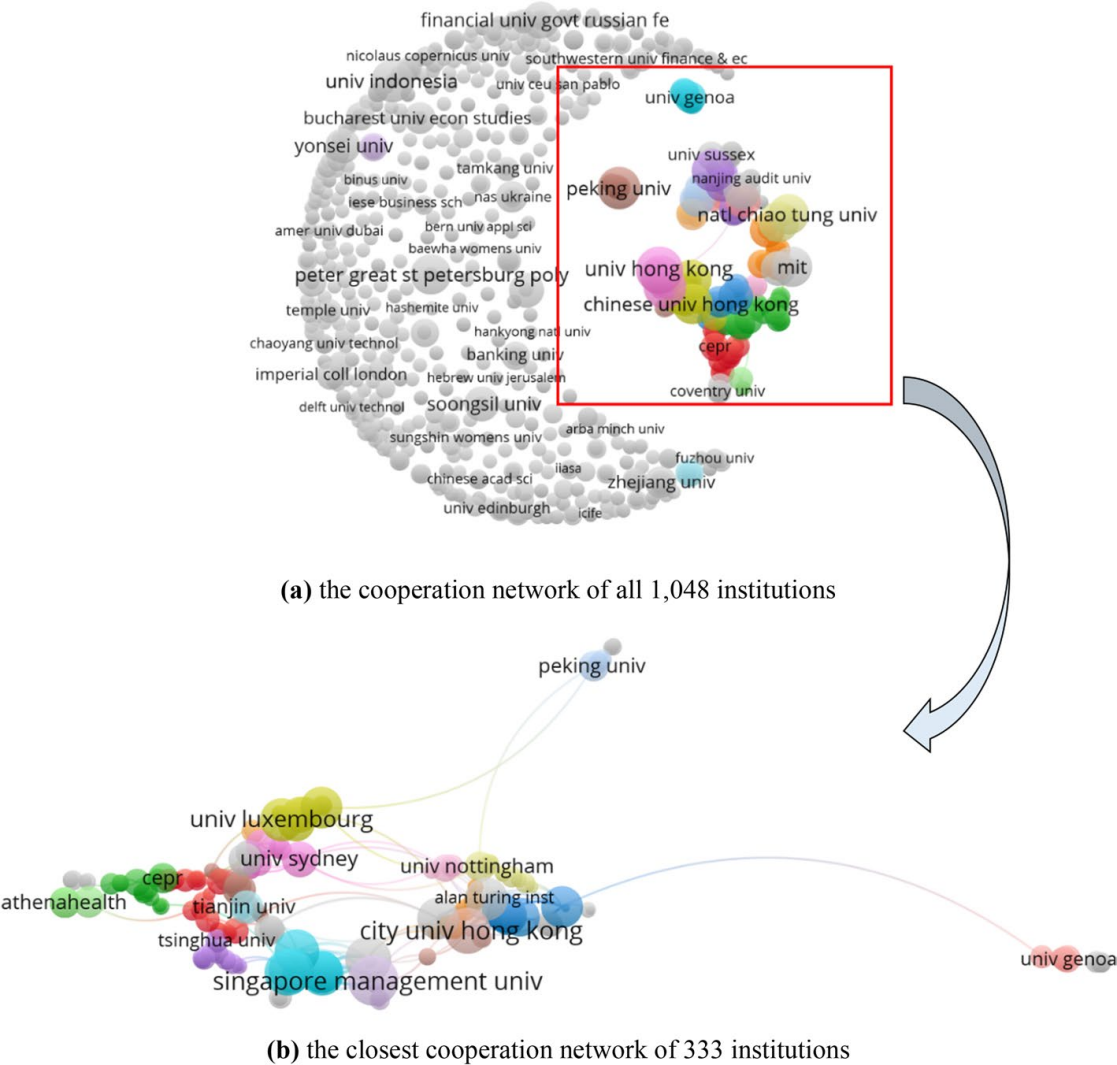
The cooperation networks of institutions (generated using Vosviewer on data)

There are 1,048 institutions in the field of FinTech. Figure 9 presents the cooperative relationship networks of all institutions and the closest network of 333 institutions. In Fig. 9, the sizes of the nodes present the number of documents. The gray nodes indicate that the articles published by these institutions in the field of FinTech were not done in cooperation with other institutions. To improve the TP and TC of publications in this area, cooperation urgently needs to enhance. In the closest cooperation network, the size of the node represents the number of total link strengths. As a result, Singapore Management University is the most cooperative institution, and its total link strength is 38 related to 8 documents with 81 citations. The University of Minnesota System (its total link strength, related documents, and citations are 32, 6, and 81, respectively), New York University (32, 6, and 77), City University of Hong Kong (32, 7, and 56), and the City University of New York (30, 4, and 61) follow. Even though they are not in the top 5 most productive institutions list, their influence is at a higher level.

### Productive journals

Table 5 lists the top 10 most productive journals and their corresponding important indicators, such as TP, TC, and impact factor (IF), ≥ 100 and ≥ 50. Considering that FinTech is a new field, the number of TP and TC is relatively low. For the top 10 list, three are proceedings papers/books. Four belong to Q1 journals, including *Electronic Commerce Research and Applications*, *Journal of Management Information Systems*, *Financial Innovation* and *IEEE Access*. Additionally, 5 journals have IF greater than 2. *Electronic Commerce Research and Applications* (its TP is 15) is the most popular journal for scholars in the field of FinTech, and it has the greatest number of TC with 248. *Financial Innovation*, as a new journal launched recently, has 8 publications related to the keywords FinTech. It has a relatively high level of TC with 80.

**Table 5:** The top 10 productive journals

Rank	Journal	IF (2019)	IF	JCR	TP	TC	C/P	≥ 100	≥ 50	The most cited articles	Time cited
1	*Electronic Commerce Research and Applications*	3.824	4.3	Q1	15	**248**	16.53	1	1	Au and Kauffman	151
2	*Advances in Social Science Education and Humanities Research*	–	–	–	10	1	0.1	0	0	Zhang and Yang	1
3	*European Business Organization Law Review*	0.431	0.443	Q4	10	32	3.2	0	0	Huang	10
4	*Journal of Management Information Systems*	3.949	5.399	Q1	9	165	**18.33**	0	1	Gomber et al	55
5	*Education Excellence and Innovation Management Through Vision 2020*	–	–	–	8	1	0.125	0	0	Maududy and Gamal	1
6	*European Journal of Finance*	1.217	1.464	Q3	8	3	0.375	0	0	Demir et al	1
7	*Financial Innovation*	2.964	–	Q1	8	80	10	0	0	Chen et al	17
8	*Advances in Intelligent Systems and Computing*	–	–	–	7	4	0.57	0	0	Bhatt et al	2
9	*IEEE Access*	3.745	4.076	Q1	7	2	0.29	0	0	Tian et al	2
10	*Electronic Markets*	2.981	4.417	Q2	6	62	10.33	0	0	Gimpel et al	16

Note: The maximum value of the relevant data is highlighted in bold

**Fig. 10:**
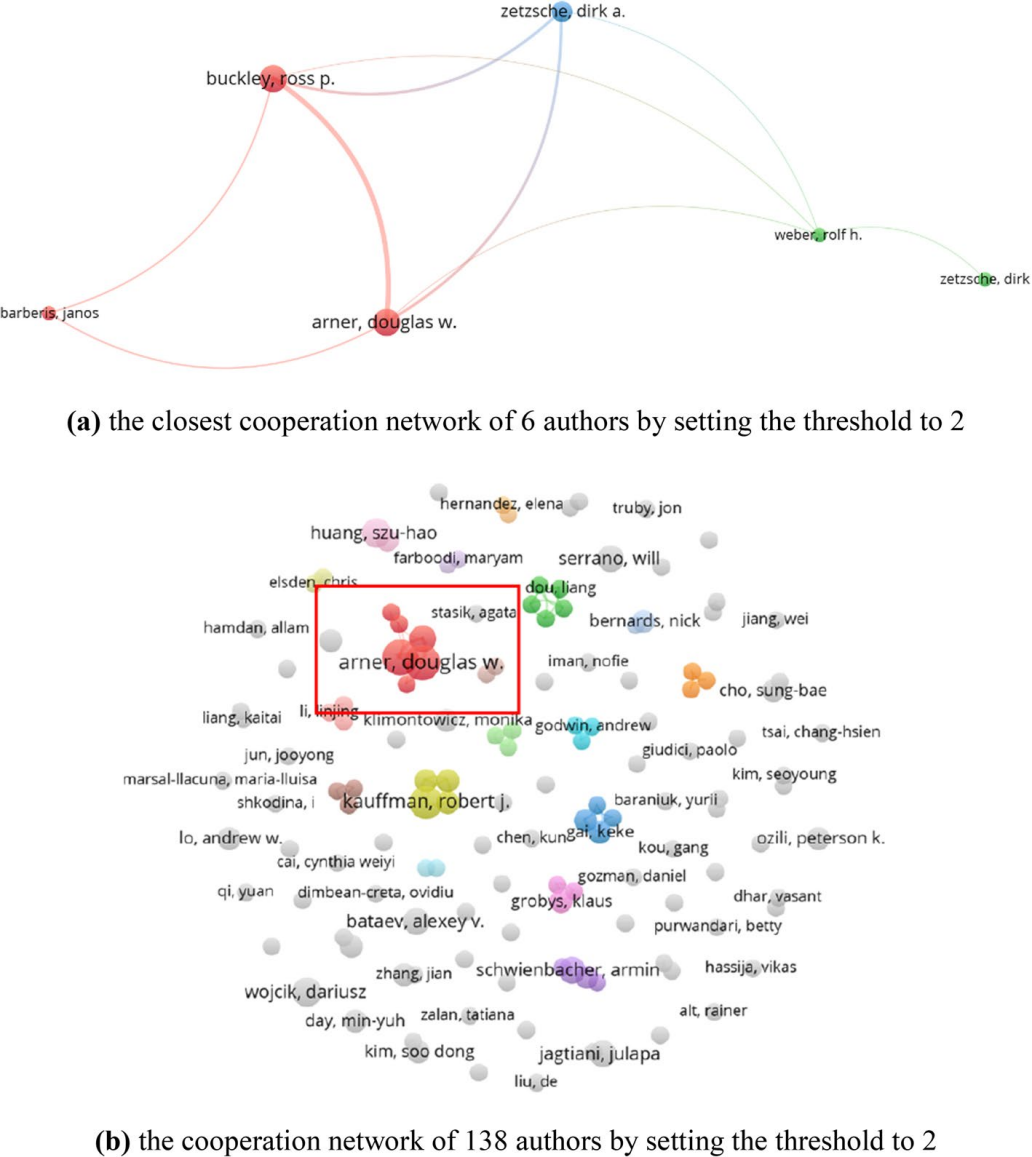
The cooperation network of authors (generated using Vosviewer on data)

Moreover, only one journal, i.e., *Electronic Commerce Research and Applications*, has published a paper cited more than 100. The paper is, *The economics of mobile payments: Understanding stakeholder issues for an emerging financial technology application*. It examined a new technology application, in association with the revolution in wireless connectivity, i.e., mobile payments (Au and Kauffman 2008). Similarly, the number of articles posted by authors is scattered. The authors who have published more than 5 papers are DW Arner, RP Buckley, RJ Kauffman, SH Huang, D Wojcik, and J Zhang. Among them, the work of RJ Kauffman has the most citation with 151, i.e., *The economics of mobile payments: Understanding stakeholder issues for an emerging financial technology application*. *FinTech, RegTech, and the Reconceptualization of Financial Regulation* (Arner et al. 2017), the work of DW Arner and RP Buckley has the most citation (38) for the documents published by them in the field of FinTech, which once again confirms the importance of cooperation. The cooperation network is shown in Fig. 10. Setting the threshold to 2 means the minimum number of documents of an author. We obtained the closest network (Fig. 9a) from the complete cooperation network (Fig. 10b). According to the above analysis, the spirit of cooperation is worth promoting to enhance the influence of publications and authors.

## Citation structure analysis

To investigate the influence of the cited authors and journals further, this section conducts citation structure analysis, including citation analysis and co-citation analysis in terms of authors and journals. Thus, scholars who are interested in this field can locate authoritative academics and journals accurately.

**Fig. 11:**
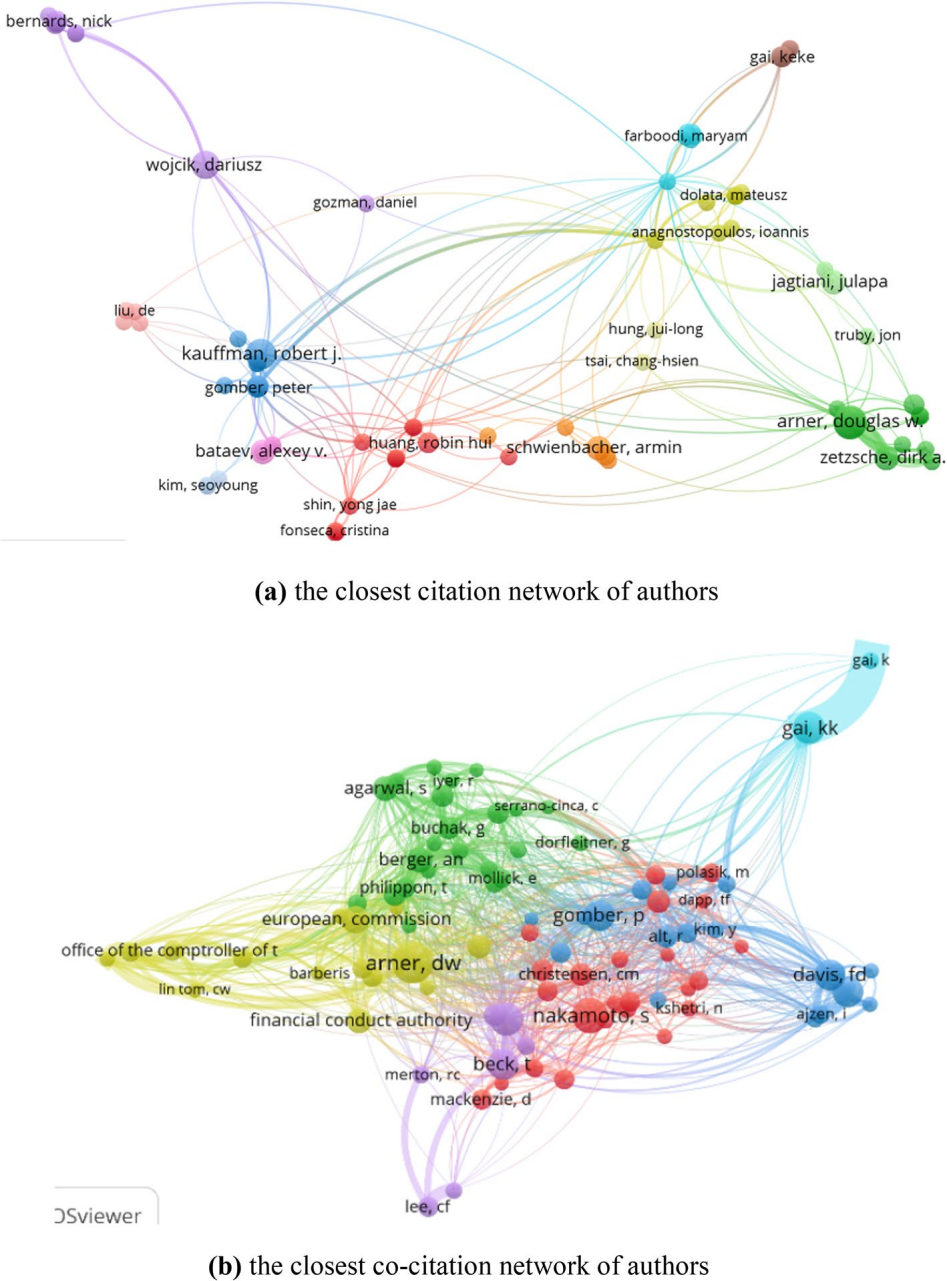
The citation and co-citation network of authors (generated using Vosviewer on data)

Citation explains the number of times the author has been cited, and co-citation reflects that two authors/journals/references/sources are cited in one paper simutaneously. According to Vosviewer, we obtained 138 authors out of 2,025 based on the minimum number of documents of an author (i.e., the threshold is 2), and 90 cited authors out of 20,877 based on the minimum number of citations of an author (i.e., the threshold is 20). The citation network and co-citation network are presented in Fig. 11. 79 of 138 cited authors constitute the closest citation network. They are divided into 7 clusters. The nodes and their sizes denote the authors and the citation degree, respectively. The greater the node, the more times the author is cited. 89 of the 90 cited authors constitute the closest co-citation network and are divided into 6 clusters. The connection between the two cited authors indicates that they appeared in one paper. The thicker the line, the more frequently the two authors appeared together.

For more details, Tables 6 and 7 presents the top 10 most cited authors and most co-cited authors based on some important indicators, including TP, TC, links (the number of the authors cited together), the total link strength (the weights of links), and cluster.

**Table 6:** The top 10 cited authors in the citation network of authors

Rank	Author	TP	TC	Links	Total link strength	Cluster
1	Kauffman, RJ	6	675	17	30	4
2	Arner, DW	7	317	17	48	2
3	Buckley, Ross P	7	283	17	48	2
4	Gomber, P	3	273	15	23	4
5	Parker, C	3	273	15	23	4
6	Weber, Bruce W	3	264	15	23	4
7	Gai, K	3	203	4	6	9
8	Lo, Andrew W	3	203	1	1	15
9	Qiu, M	3	190	4	6	9
10	Shin, YJ	2	180	17	20	1

**Table 7:** The top 10 co-cited authors in the citation network of authors

Rank	Author	C	Links	Total link strength	Cluster
1	Arner, DW	104	79	859	4
2	Nakamoto, S	82	62	216	1
3	World, Bank	73	72	492	5
4	Gai, KK	67	46	391	6
5	Beck, T	65	59	367	5
6	Davis, FD	65	53	437	3
7	Venkatesh, V	63	47	408	3
8	Gomber, P	62	79	505	3
9	Berger, An	46	55	314	2
10	Agarwal, S	44	48	409	2

The most cited paper with 158 citations is not included in Table 6 because AN Berger has only published one document in the field of FinTech. According to the results, among the authors who have published more than 2 papers, RJ Kauffman is the most influential with 6 publications and 675 citations, which is obvious in Fig. 11a. Kauffman focused on researching the FinTech revolution including mobile payment and cards, evaluating changes and transformations in different areas of financial services (Au and Kauffman 2008; Gomber et al. 2018; Kauffman et al. 2017). Additionally, DW Arner is the most co-cited author in the co-citation network (the number of citations is 104). 79 authors have been cited with him. Arner focuses on digital financial service and its regulation, sustainability features, and RegTech (Arner et al 2017, 2020; Zhou et al. 2015). Furthermore, the citation network and co-citation network of journals are illustrated. The corresponding indexes are listed in Table 8. The meanings of the nodes, sizes of the nodes, the links, and their thickness are similar to that of the authors.

**Table 8:**
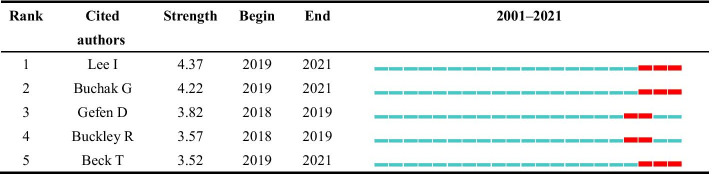
The top 5 cited authors with the strongest citation bursts from 2001 to 2021

**Fig. 12:**
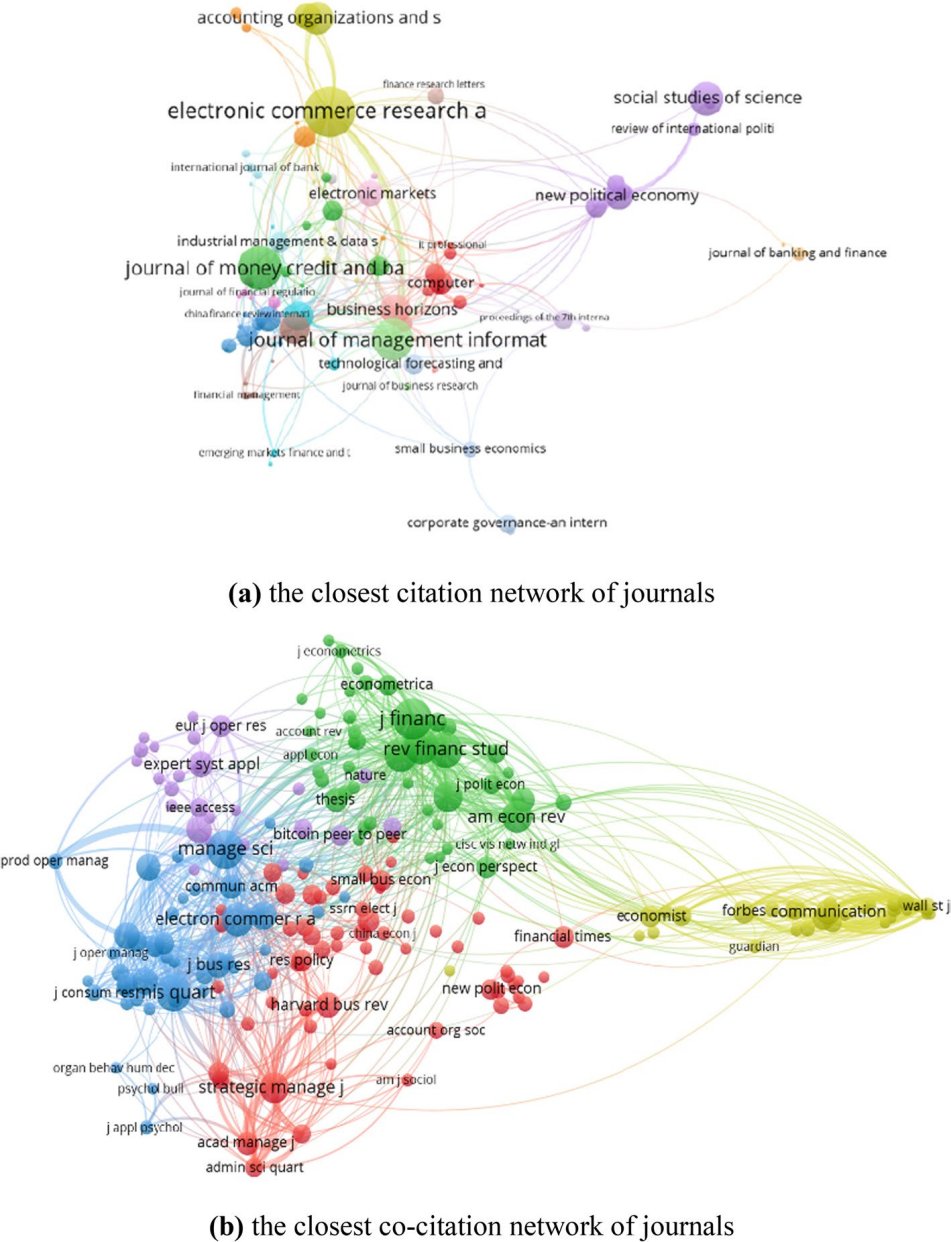
The citation and co-citation network of journals (generated using Vosviewer on data)

**Fig. 13:**
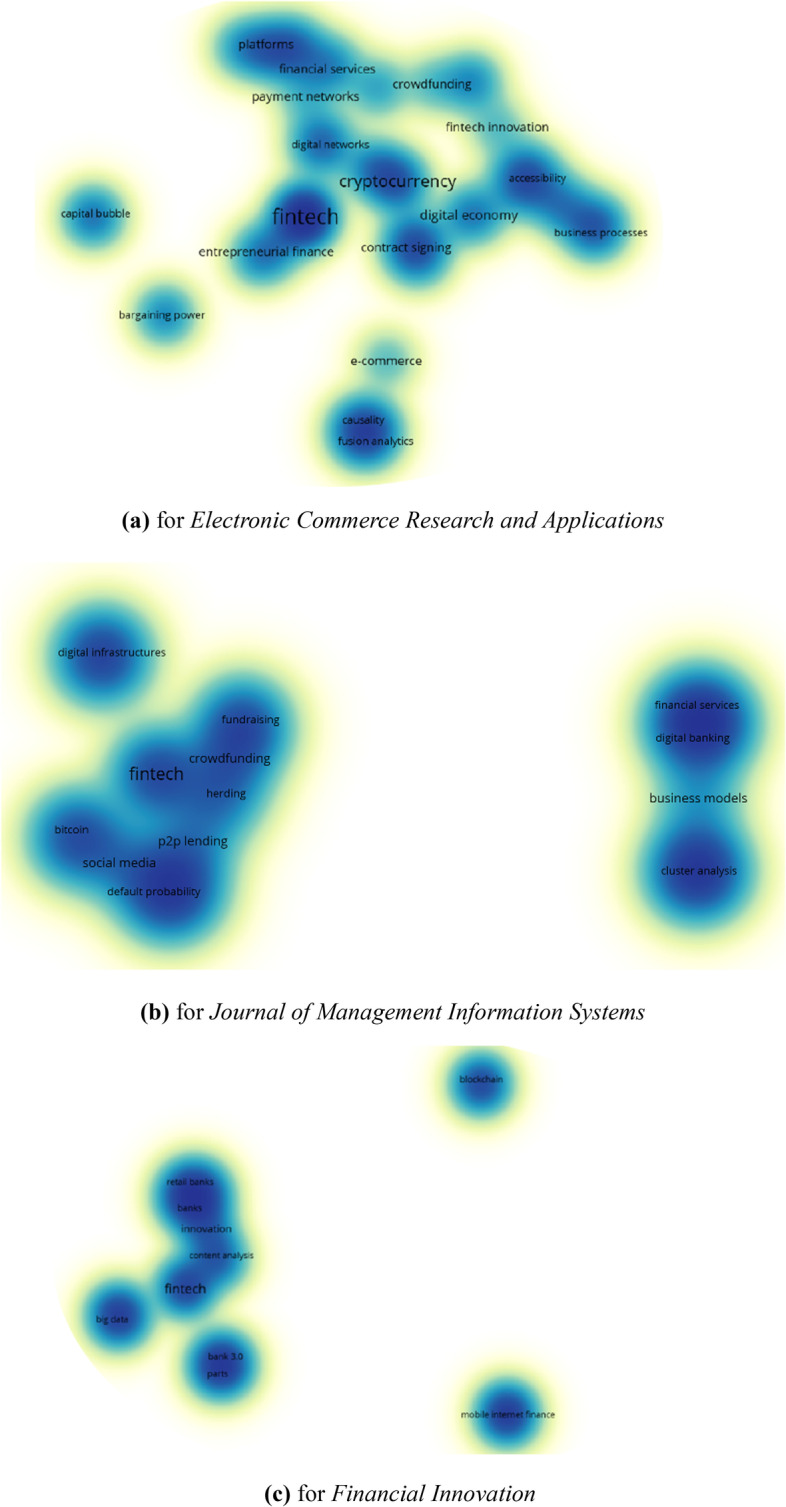
The research topics of top important journals (generated using Vosviewer on data)

From Fig. 12, the top cited journals are *Electronic Commerce Research and Applications*, *Journal of Money Credit and Banking*, *Social Studies of Science*, *Accounting Organizations and Society*, *Journal of Economics and Business*, *Business Horizons*, and *Financial Innovation*. The top co-cited journals include *Journal of Finance*, *Management Science*, *MIS Quarterly*, *Review of Financial Studies*, *Journal of Financial Economics*, *Journal of Banking & Finance*, *Electronic Commerce Research and Applications*, *American Economic Review*, and *Strategic Management Journal*. Next, combining the top citation journals with the top prolific journals (Table 5), we selected three important journals belonging to Q1 (*Electronic Commerce Research and Applications*, *Journal of Management Information Systems*, and *Financial Innovation*) to represent their research focus and help scholars conduct targeted research based on the co-occurrence analysis of author keywords, as shown in Fig. 13. We obtained 84, 50, and 33 author keywords of the three journals, respectively. Except for the general keyword, i.e., FinTech, the foci of *Electronic Commerce Research and Applications* include cryptocurrency, blockchain, digital economy, mobile payment, and bitcoin. The top hot topics of the *Journal of Management Information Systems* are business models, crowdfunding, P2P lending and bitcoin. For *Financial Innovation*, big data, blockchain, and digital banking are popular research topics. Combining with Fig. 5 and Table 3, the main research subfields are obvious.

Then, this paper conducted a the burst detection analysis (a popular method that can reflect the explosive data attracted attention by the academic in a certain period) (Xu et al. 2019; Kleinberg 2003) for the cited authors, journals, and references. It can be used to reflect the dynamic changes of publications in the field of FinTech.

**Fig. 14:**
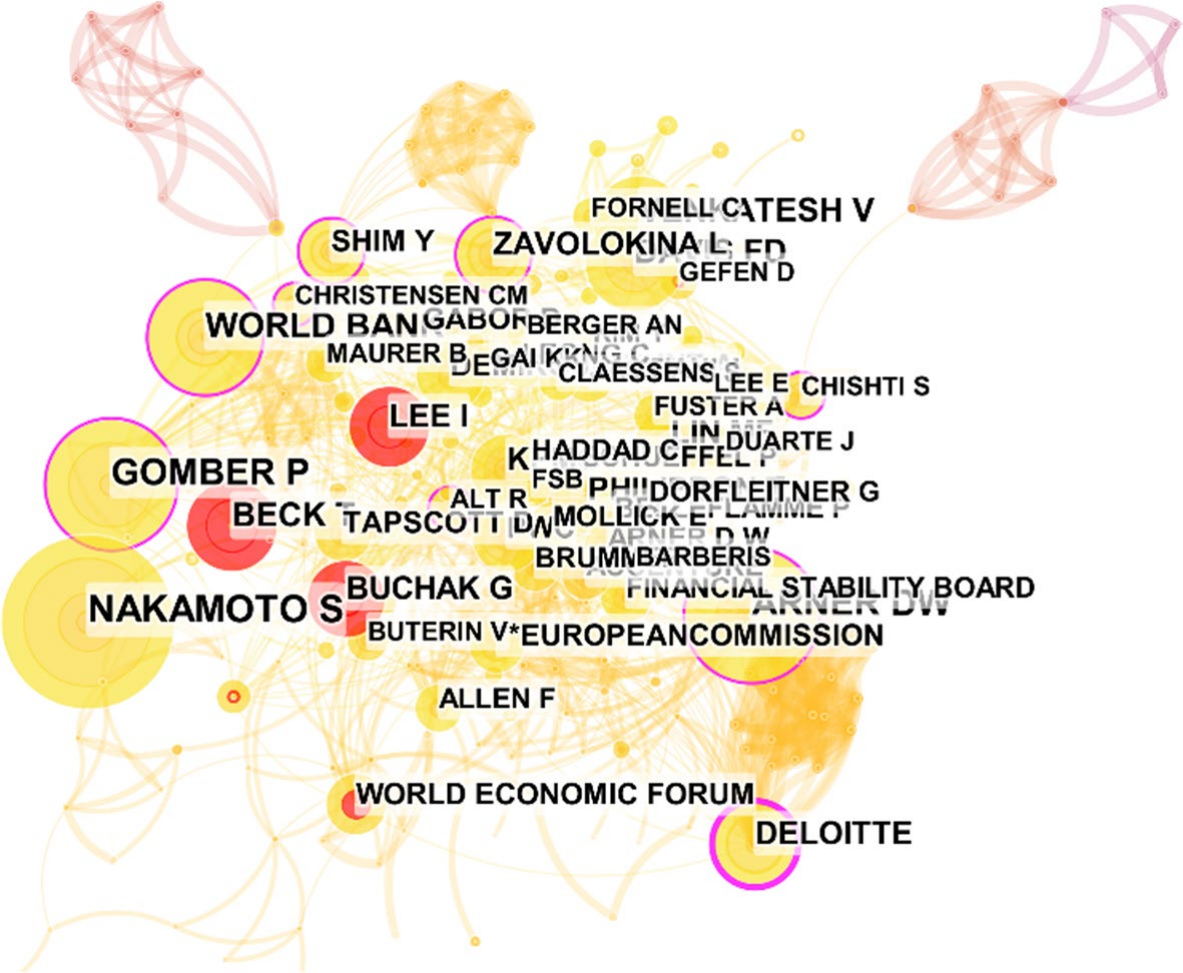
The visualization of the cited authors (generated using CiteSpace on data)

The visualization of the cited authors is shown in Fig. 14. The node and its size denote the cited authors and citation, respectively. The red nodes represent the authors with the strongest citation bursts. As a result, we obtained 6 terms with the strongest citation bursts; one of them is the name of a forum, the World Economic Forum. Thus, Table 8 lists the other 5 cited authors with the strongest citation bursts.

From Table 8, we have noticed that most of the research was done recently. Not surprisingly, only a few authors have the strongest citation bursts and are all close to the present. I Lee, G Buchak, and T Beck have been closed to 2021. Of the 848 publications, I Lee published only one paper, *FinTech: Ecosystem, business models, investment decisions, and challenges*. It introduced a historical view of FinTech; discussed the ecosystem of the FinTech sector, various FinTech business models, and investment types; and illustrated real options for FinTech investment decisions (Lee and Shin 2018). The paper, *FinTech, regulatory arbitrage, and the rise of shadow banks*, published by G Buchak, studied the contributions of regulatory differences and technological advantages to the growth of the shadow bank market (Buchak et al. 2018). FinTech has widespread applications in various fields and is still expanding.

According to CiteSpace, 14 journals have been cited frequently in a certain period (Table 9). The citation frequency for most of the journals has increased since 2016. The strength of the *Financial Times* is the strongest with a value of 8.2555. *Financial Times* is a newspaper edited in London and has a strong influence on the financial policies of the British government. For academic journals, the strength of the *Harvard Business Review* is the strongest with 5.2399. It focuses on leadership, organizational change, negotiation, strategy, operations, marketing, finance, and managing people. The citation burst of the cited journal of *Economic and Social Review* has the longest duration of 12 years from 2006 to 2017. Moreover, only one has continued until 2021, i.e., *Business Horizons*.

**Table 9:**
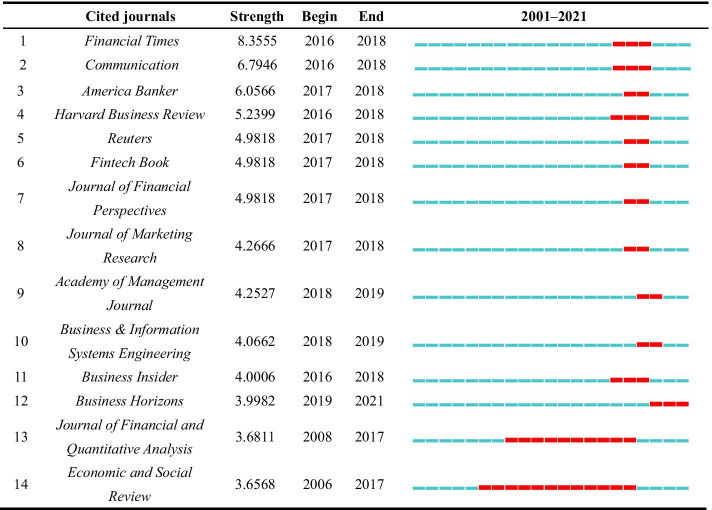
The top 14 cited journals with the strongest citation bursts from 2001 to 2021

**Fig. 15:**
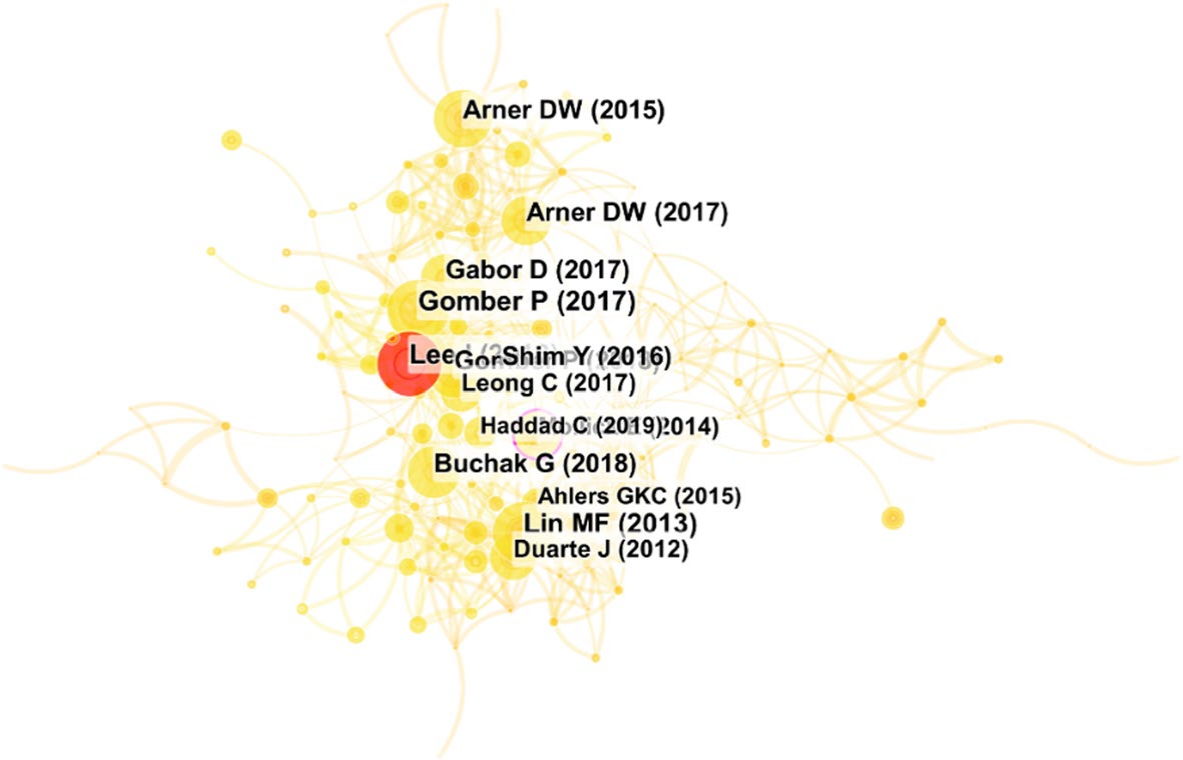
The visualization of the reference network (generated using CiteSpace on data)

Next, we studied the relationship between references related to FinTech. Based on CiteSpace, the reference network is constructed as shown in Fig. 15. Similarly, the red node is the reference with the strongest citation burst. There is only one reference with the strongest citation burst (its strength is 3.6071), i.e., *Social media analytics for enterprise: typology, methods and processes*. It provided an overview of social media analytics for managers (Lee 2018), and was published on *Business Horizons* in 2018. As listed in Table 10, the duration result begins in 2019 and continues to 2021, which reflects that its influence is continuing in the field of FinTech.

**Table 10:**
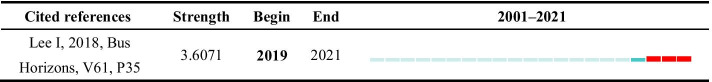
The reference with the strongest citation bursts from 2001 to 2021

**Fig. 16:**
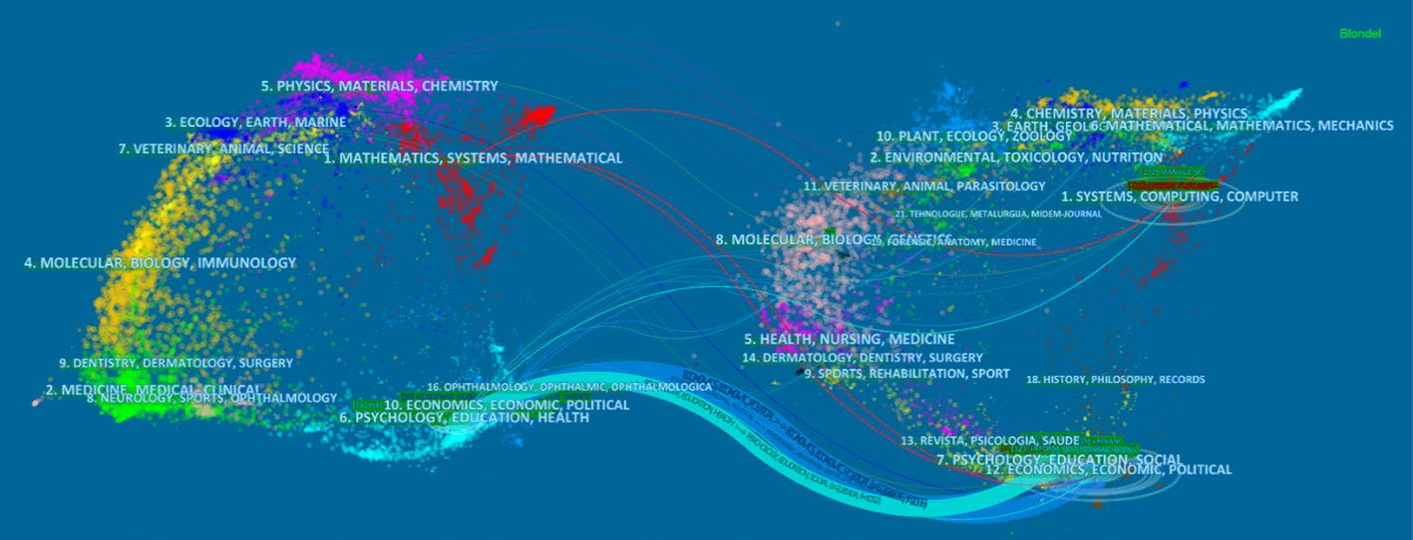
Overlay analysis of all 848 publications (generated using CiteSpace on data)

To show the trends of the documents more intuitively, Fig. 16 presents the overlay analysis (Nita 2019). It is divided into two parts, i.e., citing (left part) and cited (right part). The curves denote the citation connections. For the oval in Fig. 15, the horizontal axis and the vertical axis reflect the numbers of authors and documents, respectively. The more papers published in journals in specific fields, the longer the vertical axis. The greater the number of authors, the longer the horizontal axis. On the left, we can see that the journals mainly belong to cluster 1 (mathematics, systems, mathematical), cluster 6 (psychology, education, health) and cluster 10 (economics, economic, political). Correspondingly, the number of authors is large. For the cited part, the references are involved in many areas, for instance, chemistry, materials, physics (cluster 4), environment, toxicology, nutrition (cluster 2), molecular, biology and genetics (cluster 8). Of course, they are mainly concentrated in the same three areas as the left. In comparison, the research in the field of FinTech has a widespread impact on many fields and is still expanding.

## Discussion

FinTech is still at an early stage. Combining with the theme view, co-occurrence networks, and a timeline view of author keywords, the related research areas mainly involve lending, blockchain, machine learning, big data, financial regulation, and financial inclusion. The research not only promotes scientific and technological progress but also plays a key role in economic development. To clarify the challenges and possible opportunities reasonably in the future, this section combines the derived documents and the characteristics of the current economic environment. Subsequently, the challenges and development prospects are discussed, especially from the perspective of the impact of big data and COVID-19 on FinTech.

### Big data

As a critical technology and one of the research topics closely related to FinTech (see Fig. 6), big data has a great influence on reshaping the market by introducing new algorithmic technologies and is the key production element of the digital economy and digitalization (Gruin 2020). It is the best technical support for financial innovation. The integration of technologies such as big data and cloud computing has promoted the rapid development of the Internet of Things, which has realized the interconnection and intercommunication of people, people and things, and things and things, leading to explosive growth in the amount of data.

As the core means of production and production factors of Digital Economy 2.0, the value of data needs to be realized by the technology cluster of the supporting layer, including artificial intelligence, blockchain, and AR/VR. Data intelligence is the core of future financial (Gai et al. 2018a, b).

**Fig. 17:**
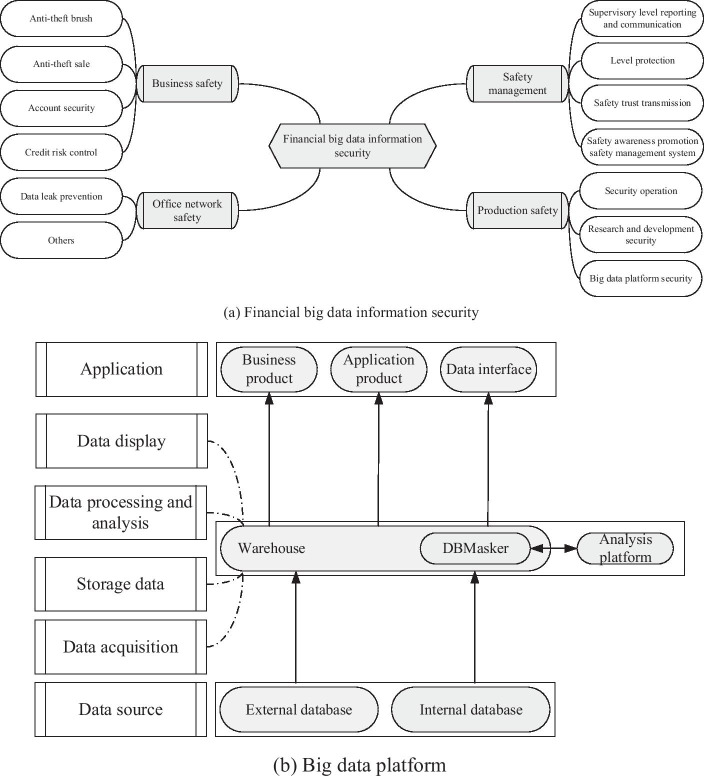
Big data security

With the development of the social economy, progressively more financial companies are beginning to build their big data platforms, from banks to P2P to insurance and securities. Ensuring data security and improving data usage efficiency, distinguishing and filtering the interfering elements, and obtaining more effective models or financial products will play a vital role (Hung et al. 2020). For example, (1) banking will analyze behavior data of clients, including deposits, withdrawals, and electronic transfers, and then conduct marketing, financial product innovation, and satisfaction degree survey to send the targeted advertising information; (2) for machine learning, good data help improve the capability to predict future situations based on known variables in the learning process (Yeh and Chen 2020). The important content of big data security and a simple big data platform is shown in Fig. 17.^1^

### Influence of COVID-19 on FinTech

For various fields of research, it is important to identify the intrinsic features of complex data and use them, not limited to financial big data (Huang et al. 2021). At present, because of the impact of the pandemic, FinTech products and services face many uncertainties and unpredictable risks because many banks and financial institutions had offered online loan application services based on remote data during the COVID-19 pandemic. Najaf et al. (2021) proposed that the COVID-19 had brought a drastic change in the key determinants of P2P lending. Chen et al. (2021) investigated the impact of FinTech products on commercial banks’ performance in China. The impact of COVID-19 on financial constraints and the moderating effect of financial technology are examined by Ling et al. (2021). The development of FinTech can alleviate the negative impact of COVID-19 on corporate financial constraints.

On one hand, the explosion of the pandemic encourages the company to review its products and progress. The importance of financial regulation is self-evident (Yi et al. 2020). On the other hand, enterprises and banks should expand current products and create new service lines to accelerate the transition to e-commerce. The transition pushes enterprises and banks to reexamine and reconstruct the digital strategies aimed to master new opportunities and digital customers.

Taking lending as an example, because of online operations, it is more difficult to obtain complete client information compared with traditional face-to-face work, which can result in many malicious fraudulent loans. This will not only affect the development of enterprises but also cause a decline in the post-loan management ability. Furthermore, from the top 5 most cited authors with the strongest citation bursts by 2021, Buchak was ranked second with research on how two forces, regulatory differences and technological advantages, work online. From the top 10 most cited authors, Arner was ranked second and presented that the financial system requires increasing the use and reliance on RegTech (Arner et al. 2017). We confirmed the importance of financial regulation in the field of FinTech. Additionally, here is a challenge for financial regulators to achieve network security and decrease current online microfinance. That is, from the perspective of financial risk, how to effectively use technology, improve supervision tools, and optimize supervision paths is another challenge.

### Research feature and development prospects

The research hotspots in the field of FinTech mainly focus on specific technologies in practice and emphasize the role of the finance field. This causes insufficient theoretical discussions and neglects the innovation of technology itself. FinTech is the integration of finance and technology, the latter pushes the development of the former. Thus, in the next stage, how to jointly promote the innovation of finance and technology and achieve deep combination is the third challenge proposed in this paper.

However, with the advancement of big data, cloud computing, artificial computing, and blockchain, FinTech still has many opportunities and broad prospects for development. The core is to introduce new elements and combine them with multiple disciplines to promote the technology level. Specifically, (1) improving the modern regulation system and highlighting risk management methods help accelerate the realization of effective financial regulation and promote the stable operation of financial institutions legally. For example, under the mobile payment and artificial intelligence environment, ensuring consumer safety and avoiding information leakage is the main task of enterprises and banks at present and in the future (Tritto et al. 2020); (2) as analyzed above, the transformation triggered by emerging technologies in the FinTech has been mainly manifested in the technology applications; however, it should dig deeper into more basic theories (Mao et al. 2019); (3) with the assistance of artificial intelligence and machine learning, studying the predictive procedures with high accuracy, stability and robustness will benefit the financial market, like predicting capital markets (Alam et al. 2020) and the stock market. Additionally, the corresponding fuzzy decision-making theories and methods are helpful (Liang et al. 2017; Xu and Wang 2016). Similarly, strengthening further integration with big data will make it easier to quantify subjective and objective indicators, such as sentiment indicators.

## Conclusions

In this paper, we have presented an overall analysis of publications in the field of FinTech up to 2020. Based on WoS, we obtained 848 publications; the first document was published in 1996. Even though this topic appeared early, with the advancement of the economy and technology, the real explosions of research occurred in 2015 (see Fig. 2). From various aspects, this paper investigated the characteristics of all publications in the field of FinTech based on visualization tools.

First, the development of FinTech benefits from common progress in many fields, such as blockchain, big data, machine learning, artificial intelligence, and digital economy (see Fig. 6). Moreover, mobile money is currently a hot topic and will continue to be one (see Fig. 7). In light of countries/regions, China has the greatest number of publications, which can be illustrated by the advanced technological environment of China, such as the convenient mobile payment and intelligent life. Meanwhile, it is expected to achieve the progress of citations (see Fig. 3 and Table 2). The research hotspots of this field are clear. It can be reflected from different angles: (1) from the top productive countries/regions (see Table 3), their research topics concentrate on the blockchain, P2P lending, financial inclusion and regulation; (2) from the popular journals (see Fig. 13), *Electronic Commerce Research and Applications*, *Journal of Management Information Systems*, and *Financial Innovation* are the top influential journals. Their research focuses on e-commerce, digital economy, blockchain, big data, and banking; (3) from the timeline view of author keywords (see Fig. 7), and it not only presents the popular topics but also illustrates their time. Furthermore, burst detection analysis and overlay analysis give impetus to the scholars who are interested in FinTech to grasp the dynamic changes more intuitively. With the overall results, the current challenges (lending, risk management, and financial regulators) and future possible research directions and extensions (introducing uncertain decision making and speeding up the connection with machine learning and big data) are discussed.

In general, the findings in this paper play a key role in the next stage and encourage scholars to conduct further studies. However, because the publications presented in this paper are limited to the WoS score database and the search keywords are related to FinTech and financial technology, the content needs to be enriched in the future. We will pay more attention to the innovation research of FinTech and its dynamic development.

## Data Availability

Data used in this paper were collected from Web of Science Core Collection.

## Footnotes

1 https://www.sohu.com/a/163585024_621613
